# Autophagy in glaucoma pathogenesis: Therapeutic potential and future perspectives

**DOI:** 10.3389/fcell.2022.1068213

**Published:** 2022-12-14

**Authors:** Min Li, Zhao-Lin Gao, Quan-Peng Zhang, Ai-Xiang Luo, Wei-Ye Xu, Tian-Qi Duan, Xu-Peng Wen, Ru-Qi Zhang, Ru Zeng, Ju-Fang Huang

**Affiliations:** ^1^ Department of Anatomy and Neurobiology, School of Basic Medical Sciences, Central South University, Changsha, China; ^2^ Key Laboratory of Brain Science Research & Transformation in Tropical Environment of Hainan Province, Hainan Medical University, Haikou, China; ^3^ Anatomy Laboratory, Hainan Medical University, Haikou, China; ^4^ Transplantation Center, The Third Xiangya Hospital, Central South University, Changsha, China

**Keywords:** autophagy, glaucoma, therapeutic strategies, retinal ganglion cells, neurodegenerative

## Abstract

Glaucoma is a common blinding eye disease characterized by progressive loss of retinal ganglion cells (RGCs) and their axons, progressive loss of visual field, and optic nerve atrophy. Autophagy plays a pivotal role in the pathophysiology of glaucoma and is closely related to its pathogenesis. Targeting autophagy and blocking the apoptosis of RGCs provides emerging guidance for the treatment of glaucoma. Here, we provide a systematic review of the mechanisms and targets of interventions related to autophagy in glaucoma and discuss the outlook of emerging ideas, techniques, and multidisciplinary combinations to provide a new basis for further research and the prevention of glaucomatous visual impairment.

## 1 Introduction

Glaucoma is the most common irreversible eye disease, the second leading cause of blindness, and one of the four leading causes of moderate and severe vision impairment (MSVI) GBD 2019 Blindness and Vision Impairment Collaborators;Vision Loss Expert Group of the Global Burden of Disease Study, 2021. The number of people (aged 40–80 years) with glaucoma worldwide is expected to reach 111.8 million by 2040 (Tham et al., 2014). Recently, attention has been paid to the pathogenesis of glaucoma, providing novel insight into therapeutic strategies. Glaucoma mainly affects the retina and optic nerve. The optic nerve originates from the diencephalon and is part of the central nervous system (CNS), combined with the pathological characteristics of glaucoma, which is classified as a neurodegenerative disease. Current glaucoma treatments are based on lowering intraocular pressure (IOP) through pharmacological and surgical control. These treatments can prevent the loss of retinal ganglion cells (RGCs) and visual impairment in the early stages of the disease to some extent. Unfortunately, as visual impairment is a late symptom of glaucomatous optic neuropathy (2021), most patients with visual impairment have experienced significant RGC loss by the time of diagnosis, rendering conventional treatments futile at later stages of the disease.

Autophagy is a highly conserved catabolic pathway in organisms that promotes the degradation and recycling of cellular components and protects damaged cells by removing excess/impaired organelles and metabolites from the cytoplasm, regulating the constant renewal of peroxisomes, mitochondria, and endoplasmic reticulum, and performing subcellular level remodeling (He et al., 2019). Autophagy has recently attracted considerable attention as a vital mechanism for maintaining neuronal homeostasis, and defects in autophagy have been implicated in various neurodegenerative disorders (Frake et al., 2015; Menzies et al., 2017). Autophagy dysfunction has been implicated in chronic neurodegenerative diseases such as Alzheimer’s disease (Nixon et al., 2005), Parkinson’s disease (PD) (Pan et al., 2008), and Huntington’s disease (Ravikumar et al., 2004) and in acute diseases such as cerebral hypoxia/ischemia and trauma (Wong and Cuervo, 2010). Autophagy plays a key regulatory role in the development and progression of glaucoma and is a potential new therapeutic target. In recent years, numerous studies have demonstrated that autophagy plays an important role in the development of glaucoma and that dysfunctional autophagy is closely associated with this disease (Villarejo-Zori et al., 2021). In addition, glaucoma is thought to be associated with mutations in autophagy-related genes (Mizushima and Levine, 2020). Autophagy is a particularly crucial 'clean-up station' in neurons efficiently removes misfolded proteins and damaged or aged organelles to safeguard cellular homeostasis (Chen et al., 2019); if not removed effectively, these waste products can accumulate and lead to neuronal degeneration and death (Chen et al., 2019), causing permanent damage to the retina. Research on autophagy in glaucomatous optic nerve injury mainly focuses on organismal stress in response to high IOP (HIOP), optic nerve protection, immune regulation, trabecular meshwork (TM) dysfunction, and scar modulation (Ishikawa et al., 2021). These studies suggest that autophagy is dysfunctional in glaucoma, i.e., impaired autophagy is part of the pathogenesis.

Herein, this review focuses on the role of autophagy in the pathogenesis of glaucoma and the therapeutic potential of targeting the autophagy pathway. The normal function of autophagy is first described first, followed by a discussion of the major roles of autophagy in some of the pathogenic mechanisms of glaucoma. Finally, the current and promising approaches to modulating autophagy for glaucoma treatment are highlighted, to provide a new basis for further investigation and the prevention of glaucomatous visual impairment.

## 2 The function and mechanism of autophagy

### 2.1 Classification and function of autophagy

**FIGURE 1:**
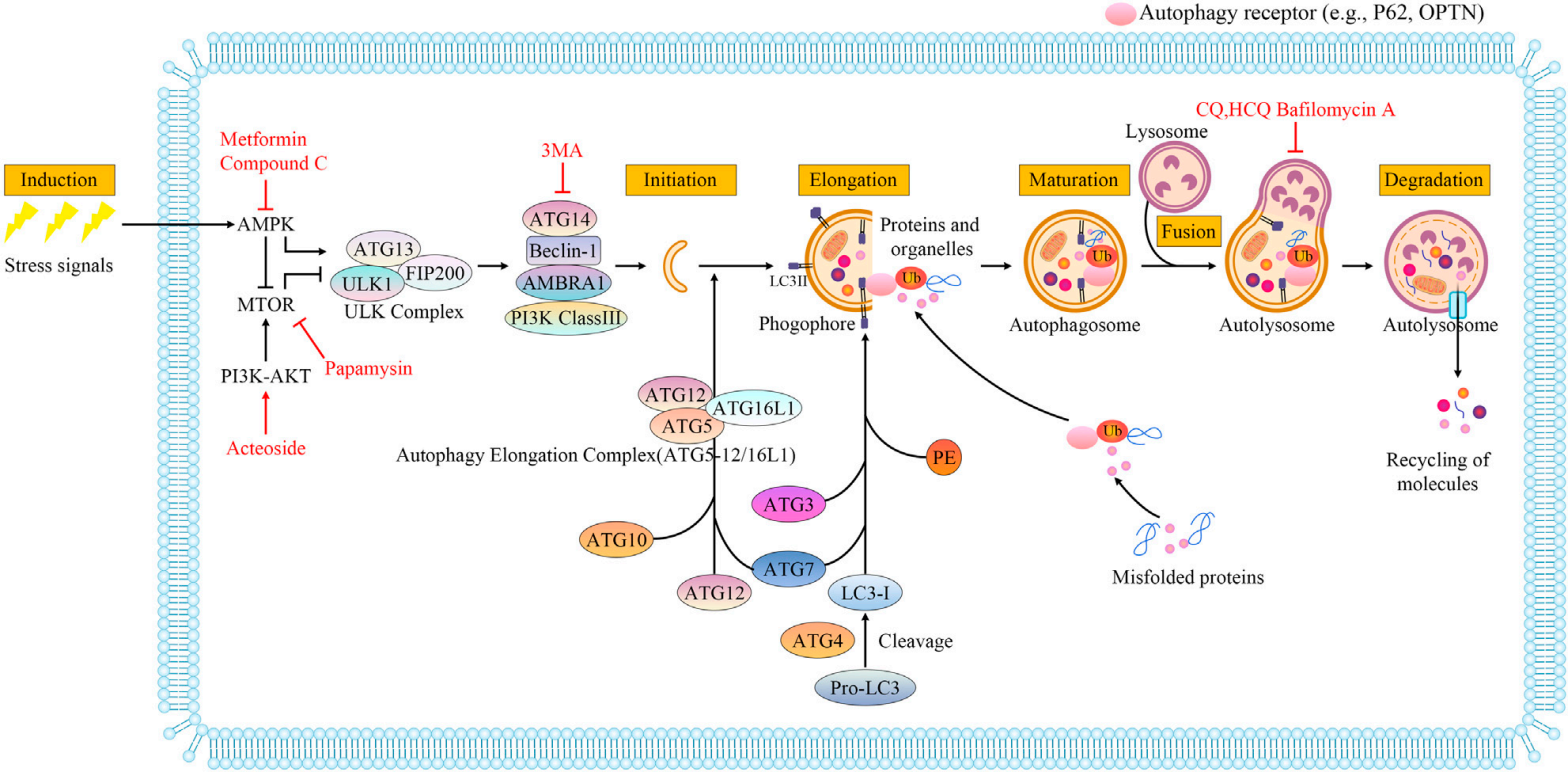
Molecular mechanisms during macroautophagy processes. Autophagy is a multistep process that includes induction, initiation, elongation, maturation, fusion with lysosomes, and degradation. The autophagy process is regulated by the mTOR and AMPK signaling pathways, which are responsible for monitoring the nutritional status of the cell. AMPK is an activator and mTOR acts as an inhibitor through the phosphorylation of ULK1 at distinct residues. The ULK1-containing initiation complex triggers phagophore formation by phosphorylating components of the class III Ptdlns3K nucleation complex. During autophagosome biogenesis, after ATG5 binds ATG12, the ATG5-ATG12 conjugate binds to ATG16L1 to generate the autophagy elongation complex (ATG5-12/16L1), which regulates lipidation of LC3. LC3, a ubiquitin-like modifier, is cleaved by the protease ATG4 to form LC3-I. LC3-I conjugates with PE to form LC3-II, which incorporates into autophagosomal membranes and is critical for the formation of autophagosomes. Autophagy receptor (e.g., P62, OPTN) serves as a link between LC3 and ubiquitinated proteins or organelles targeted for degradation. Autophagosomes are eventually fused with lysosomes to form autolysosomes. The cargos in autolysosomes are degraded by lysosomal enzymes, and nutrients and metabolites are recycled. Metformin induces autophagy by targeting AMPK. MTOR signals can be inhibited directly by rapamycin. PI3K and AKT are elevated by acteoside. 3-Methyladenine(3-MA) can specifically block autophagosome formation. BAFA1 inhibits the acidification of the autolysosome by blocking the V-ATPase while chloroquine (CQ) and 3-hydroxychloroquine (HCQ) impair the maturation of autolysosomes. Drugs are depicted in red font.

The word “autophagy” is derived from Greek, meaning “self-eating” and refers to the metabolic process of cellular degradation and the recycling of cellular components within lysosomes (Galluzzi et al., 2017). There are three subtypes of autophagy, macroautophagy, chaperone-mediated autophagy, and microautophagy, which are divided depending on the mechanism by which the material destined for degradation enters the lysosome. Macroautophagy is accompanied by the emergence of a phagophore (a small bilayer of membranes) in the cytoplasm. Proteins and organelles are then encapsulated in a bilayer membrane structure that closes to form an organelle referred to as the autophagosome, which later fuses with the lysosome to enable the degradation of the enclosed material by lysosomal hydrolases (Figure 1). In chaperone-mediated autophagy, heat shock cognate 70 and several accessory chaperone proteins specifically recognize the KFERQ-like region and are transported to the lysosome for degradation (Kaushik and Cuervo, 2018). In microautophagy, the least characterized form of autophagy, the cytoplasmic cargo is destined for degradation through lysosomal or late endosomal membranes that directly engulfs cytoplasmic cargo (Schuck, 2020). Macroautophagy (hereafter referred to as autophagy) is the most widely studied of the three forms.

Although autophagy was originally thought to be a nonselective process, recent evidence suggests that it is highly selective (Villarejo-Zori et al., 2021). This selectivity means that specific types of cytoplasmic cargos have been identified (Johansen and Lamark, 2020) that are transported to the autophagosome for degradation, where they bind LC3 (light chain 3 protein) *via* LC3-interacting regions. Depending on the nature of the specific cargo, selective autophagy can be classified as heterogeneous phagocytosis (pathogen degradation), lipophagy (lipid droplet degradation), or aggregative phagocytosis (protein aggregate degradation) (Johansen and Lamark, 2020). Similarly, organelle-specific autophagy can be divided into endoplasmic reticulum, peroxisomal, mitochondrial and lysosomal autophagy (Johansen and Lamark, 2020). Recent studies have shown that the Golgi apparatus may degrade proteins by retrograde trafficking to the ER, suggesting Golgi might be a target for selective autophagy degradation (Benyair et al., 2022) (Nthiga et al., 2021).

### 2.2 Regulatory mechanisms of autophagy

Autophagy is primarily a cellular stress response and plays an important role in biological cells. Depending on the autophagy state, theprocess can be divided into several sequential stages: induction, initiation/vesicle nucleation, phagophore formation, elongation, autophagosome formation, maturation/fusion, and degradation (Kiriyama and Nochi, 2015).

Autophagy regulation is dependent on all phases. Autophagy occurs at a low basal level under physiological conditions, but it can be rapidly induced in certain states of cellular stress, such as hypoxia, glucose deficiency (Kim et al., 2011), starvation (Kuma et al., 2004) protein aggregates (Kraft et al., 2010), damaged organelles (e.g. mitochondria, endoplasmic reticulum, and peroxisomes) and intracellular pathogens infections (Glick et al., 2010). Autophagy is induced by these stresses and is regulated extensively by posttranslational mechanisms. Among all stresses, a lack of nutrients (especially amino acids) is the most important factor in the induction of autophagy. This process is mediated by upstream regulators of autophagy including mTOR and AMPK, which are responsible for monitoring nutritional status (Wong et al., 2013; Parzych and Klionsky, 2014). Phagophore formation is a key step that follows autophagy induction and is important for all phases. Autophagy initiation is controlled in part by the ULK complex (autophagy related gene (ATG)13, ULK1, FIP200), which is regulated by cellular energy sensors (AMPK and mTOR) (Bressan and Saghatelyan, 2020). After the autophagy-initiating kinase ULK1 is phosphorylated, a phagophore forms *via* phosphatidylinositol 3-phosphate (PI3P) generation (Metaxakis et al., 2018). Elongation is a crucial step in the complete autophagosomes (Ebrahimi-Fakhari et al., 2012). Tow ATG 7-catalyzed ubiquitin-like reactions control the elongation, one is to form autophagy elongation complex (ATG5-12/16L1), and the other is phosphatidylethanolamine (PE) -LC3I conjugated to form LC3-II, (Ebrahimi-Fakhari et al., 2012). Membrane elongation closure to maturation, which fuse with lysosomes to form autolysosomes and degrade proteins and organelles (Yim and Mizushima, 2020). Molecular mechanisms during macroautophagic processes are shown in Figure 1.

Autophagy is primarily regulated at the posttranslational level; various stressors can induce autophagy, and stress can also stimulate the upregulation of autophagy genes (Feng et al., 2015). The transcription factor EB (TFEB) is the main regulator of autophagy genes (Puertollano et al., 2018). Phosphorylation retains TFEB in the cytoplasm, but when dephosphorylated by stress, TFEB translocates to the nucleus where it promotes the transcription of its target genes (Puertollano et al., 2018). Optineurin (OPTN) is a gene linked to glaucoma (Shen et al., 2011), and its encoded protein is an autophagy receptor, which targets specific cargo for degradation and is itself degraded by autophagy (Mijaljica et al., 2012; Klionsky et al., 2021). Recently, at least 42 genes associated with the autophagy pathway (Mizushima, 2020), and more genes will be identified as research progresses.

### 2.3 Characteristics of autophagy in neurodegenerative diseases

Autophagy levels decline during normal human aging in all tissues examined to date, including the brain and retina, which contributes to the pathogenesis of several neurodegenerative diseases, such as glaucoma (Boya, 2017; Wong et al., 2020). Altered selective autophagy also has important pathological implications in the pathogenesis of neurodegenerative diseases. For example, inadequate removal of pathogenic protein aggregates is associated with neurodegenerative diseases (Conway et al., 2020), and dysregulation of mitochondrial autophagy, i.e., the selective removal of unnecessary or damaged mitochondria is impaired, constitutes a severe redox imbalance that is detrimental to neurons (Teodorof-Diedrich and Spector, 2018) and is common in neurodegenerative diseases.

## 3 Glaucoma pathogenesis and autophagy regulation

**FIGURE 2:**
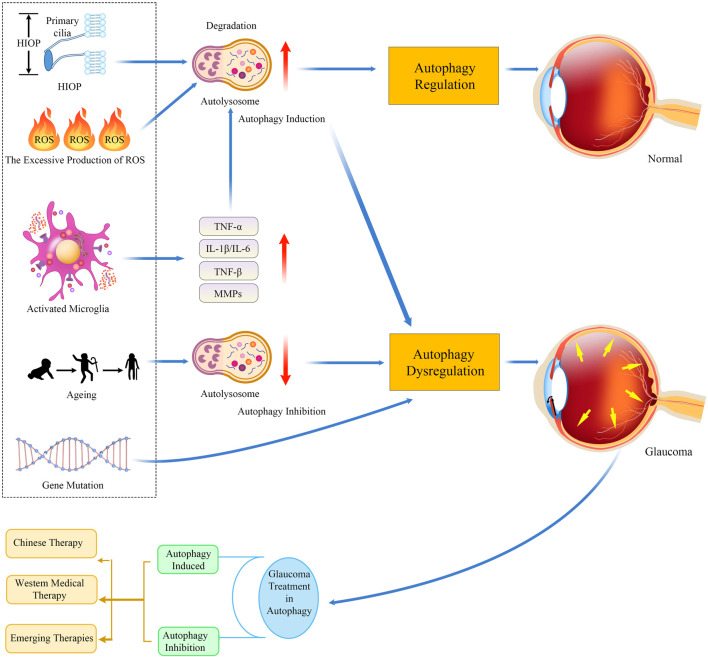
Timeline of autophagy regulation in glaucoma pathogenesis and treatment. The underlying mechanisms of glaucomatous neurodegeneration involve multiple factors, including HIOP, mitochondrial dysfunction, oxidative stress, immune-mediated neuroinflammatory responses, aging, and gene mutation. Strategies targeting the known mechanisms of autophagy might be neuroprotective and, thus, work for treating glaucoma. Abbreviations: HIOP, high intraocular pressure; TNF-α, tumor necrosis factor-alpha; IL-1β, interleukin-1 beta; IL-6, interleukin-6; TGF-β, transforming growth factor-β; MMP, matrix metalloproteinase.

Glaucoma is a multifactorial progressive optic neuropathy characterized by axonal degeneration and loss of RGCs, leading to severe and irreversible blindness (Gupta and Yücel, 2007; Tham et al., 2014). Studies have shown that the underlying mechanisms of glaucomatous neurodegeneration are not the result of a single factor, but rather a causal and interactive effect of various factors, including HIOP (mean IOP increases with age) (Bonomi et al., 1998), oxidative stress (Kimura et al., 2017), neurotrophic factor deprivation (Harvey et al., 2012), mitochondrial dysfunction (Ito and Di Polo, 2017), autoimmune dysregulation (Ito and Di Polo, 2017), age (Ko et al., 2016), and gene mutation. HIOP and aging are considered the main risk factors for glaucoma (Chang and Goldberg, 2012) (Weinreb and Khaw, 2004). Previous studies have suggested that autophagy is involved in the pathophysiology of glaucoma along with these key pathogenic triggers and is closely related to glaucoma pathogenesis. We next elaborate on the pathological mechanisms of autophagy and HIOP, mitochondrial dysfunction, oxidative stress, aging, and neuroinflammation in glaucoma (key molecules are depicted in Table 1 and Figure 2).

**TABLE 1:** Glaucoma pathogenesis and autophagy regulation.

Patterns	Key molecules/pathways of pathogenesis	The role of autophagy	Regulation of autophagy pathways	Ref
HIOP	Distention, stretching, and finally malfunction of the TM cells, resulting in persistent HIOP.	Maintaining IOP homeostasis	HIOP --- Sensing PC of TM cells ---Transduction of AKT1 and SMAD2/3 signaling --- Autophagy induction ---Maintain IOP homeostasis	Shim et al. (2021)
Mitochondrial dysfunction, oxidative stress	Excessive production and accumulation of ROS, resulting in oxidative damage, which leads to mitochondrial dysfunction and injury of RGCs which have a high density of mitochondria	Eliminating oxidized cellular components and regulating cellular ROS levels to maintain cell health	ROS --- Activation of transcription factors (HIE-1α, NRF2, P53, and FOXO3) --- Post translational regulation (oxidation, phosphorylation) --- Gene expression required for autophagy induction (BECN1, LC3, SQSTM1, etc.) --- Autophagy induction	Chang et al. (2022)
Immune-mediated neuroinflammatory responses	Chronic inflammation may translate into RGC degeneration (common mediators of neuroinflammation in glaucoma including TNF-α, IL-1 β/IL-6, TGF-β, MMP-2/MMP-9, C1q)	Guiding microglia activation toward the anti-inflammatory M2 phenotype	Pro-inflammatory cytokine TNF-α ---Activation of AKT/mTOR signaling ---Inhibition of autophagy flux and driving microglia shift toward the M1 phenotype	Quaranta et al. (2021)
Aging	Glaucoma is a disease of the aging eye, and its prevalence increases with age	Autophagy declines with age, which can be detrimental to cells	Aging --- Dampened AMPK, enhanced mTOR signaling, and decreased Beclin 1 expression --- The extent of autophagy decreases	(Bell et al., 2020) (Madhu et al., 2022)
Gene mutation	The gene mutation of OPTN, TBK1, and MYOC.	Autophagy removes beneficial content or fails to remove harmful one	Gene mutation --- Impaired autophagy --- TFRC degradation or the disruption of molecules degradation (e.g., TDP43)	Zhang et al. (2021b)

HIOP, high intraocular pressure; TM, trabecular meshwork; PC, primary cilia; IOP, intraocular pressure; ROS, reactive oxygen species; RGCs, retinal ganglion cells; HIF-1α, hypoxia-inducible factor-1α, NRF2, nuclear factor erythroid 2–related factor 2, FoxO3, forkhead box O-3, LC3, light chain 3 protein; TNF-α, tumor necrosis factor-alpha; IL-1β, interleukin-1 beta; IL-6, interleukin-6; TGF-β, transforming growth factor-β; MMP, matrix metalloproteinase; AMPK, AMP-activated protein kinase; OPTN, optineurin; TBK1, TANK binding kinase 1; MYOC, myocilin; TFRC, transferrin receptor.

### 3.1 Pathological IOP elevation and autophagy in glaucoma

Autophagy activation in HIOP (acute or chronic) has been observed in different experimental models of glaucoma (Piras et al., 2011; Hirt et al., 2018). IOP is exerted by the aqueous humor (AH) on the inner surface of the anterior segment of the eye; normal is approximately 16 mmHg (Carreon et al., 2017). AH is a clear fluid produced by filtering blood through the ciliary body, which enters the anterior chamber of the eye at a relatively constant rate. AH nourishes the cornea, lens, and TM in the anterior chamber and exits mainly through the TM/Schlemm’s canal (SC). Elevated IOP is caused by the failure of the TM/SC, a conventional outflow tract, leading to an inability to maintain an adequate level of AH outflow resistance (i.e., impaired outflow) (Stamer and Acott, 2012). Thus, the TM is therefore particularly important for maintaining normal IOP as it is the main outflow pathway for AH. Dysregulation of autophagy in the retina and TM has been demonstrated in the HIOP model of glaucoma (Nettesheim et al., 2020).

Numerous studies have demonstrated that cells in the outflow pathway are subjected and responsive to fluctuating IOP and mechanical loads (Hirt and Liton, 2017). Eleven known mechanotransduction channel proteins were detected in TM tissue and isolated TM cell cultures (Tran et al., 2014). Experiments conducted in *in vitro* perfusion eyes have demonstrated the presence of a mechanism regulating IOP homeostasis in TM/SC outflow pathway tissues (Acott et al., 2014). After the perfusion rate increased, resulting in a short IOP increase, even if the inflow was maintained at high pressure, the IOP gradually returned to the baseline level in a few days (Bradley et al., 2001). This observation has been confirmed by various researchers, suggesting that a compensatory mechanism helps the maintenance of IOP homeostasis (Borrás, 2003; Baetz et al., 2009; Porter et al., 2014). Cells in the outflow channel sense pressure imbalance in the form of mechanical stretching, which is thought to trigger a response (Hirt and Liton, 2017). TM cells are prone to stretch injury after long-term exposure to tension; thus, they must be guided to adapt to this mechanical force and repair the damage caused by potential stretching. One study suggested that autophagy may be a possible cellular adaptation mechanism in response to this stretch (Shim et al., 2020). A recent study has shown that primary cilia (PC) are mechanosensory for stretch-induced autophagy in TM cells, and PC-mediated autophagy is critical for IOP homeostasis (Shim et al., 2021). Stretch-induced autophagy under normal physiological forces is helpful to eliminate damaged components and accelerate the renewal of proteins and organelles, resulting in increased cytoskeletal and cortical stiffness in TM cells (Liton, 2016), maintaining cellular homeostasis, and inhibiting cell death (Porter et al., 2014). Conversely, autophagy dysregulation may trigger cell death and lead to disease if the mechanical forces exceed the physiological limits or if the conditions are pathological (Hirt and Liton, 2017). Overactivation of autophagy is a potential cellular mechanism leading to optic nerve degeneration in DBA/2J mice (Hirt et al., 2018).

### 3.2 Mitochondrial dysfunction, oxidative stress, and autophagy

Mitochondria are exquisitely dynamic organelles that move along axons and dendrites in neurons and constantly respond to endogenous and exogenous stimuli, to satisfy the energy requirements of neuronal networks and to maintain neuronal Ca2+ homeostasis and synaptic plasticity (Mattson et al., 2008). The highly coordinated regulation of mitochondria is essential for maintaining the energy in RGC dendrites and axon terminals for optimal neurotransmission (Ito and Di Polo, 2017). To date, several studies have shown that oxidative stress damage and mitochondrial dysfunction are involved in the pathogenesis of glaucoma and are tightly linked to autophagy pathways (Chang et al., 2022).

Studies have shown that overexpression of oxidative stress markers including lipid peroxidation, and oxidative DNA damage is present in various ocular tissues in glaucoma patients (Fernández-Durango et al., 2008). Oxidative stress results from the overproduction of reactive oxygen species (ROS) (⋅O_2_−, ⋅OH, and H_2_O_2_), which are byproducts of cellular respiration, particularly during mitochondrial oxidative phosphorylation, and are produced by the electron transport chain in the inner mitochondrial membrane (Shadel and Horvath, 2015). Although low ROS at physiological functions in redox signaling, excessive ROS produced by mitochondrial malfunction can lead to the oxidative damage of cellular components, comprising lipids, proteins, and DNA (Shim and Liton, 2022). One is in the mitochondria ROS in the form of superoxide and H_2_O_2_ mediates the sustained activation of nuclear factor kappa-B (NF-κB) and the upregulation of inflammatory markers like interleukin-1α (IL-1α) and endothelial leukocyte adhesion molecule-1 (ELAM-1) (Li et al., 2007). Another crucial source is the production of ROS through the iron-catalyzed Fenton/Haber-Weiss reaction (Kruszewski, 2003; Yauger et al., 2019). The Fenton/Haber-Weiss reaction results in the production of highly oxidized hydroxyl radicals, which induce lipid and protein peroxidation and cause oxidative damage to DNA (Kruszewski, 2003). Among other things, the increase in lipid peroxidation may be attributed to a deficiency in the antioxidant system (Fernández-Durango et al., 2008). ROS exert two regulatory effects on autophagy, inducing autophagy through the activation of transcription factors (HIF-1α, NRF2, P53, and FOXO3) and by driving the expression of genes required for autophagy induction (including BECN1, LC3, SQSTM1) *via* posttranslational regulation (Chang et al., 2022). Autophagy can also be inhibited by the oxidation of ATG proteins (ATG7 and ATG10) or by the inactivation of autophagy regulators (TFEB and PTEN) (Su et al., 2018; Chang et al., 2022). In turn, autophagy can reduce ROS levels and alleviate oxidative damage by eliminating damaged molecules (e.g., protein aggregates) and damaged organelles (e.g., mitochondrial cartilage), thus maintaining proper redox signaling and DNA damage repair systems (Li et al., 2015). These findings suggests that mitochondrial autophagy induction may improve the pathogenesis of glaucoma.

### 3.3 Immune-mediated neuroinflammatory responses and autophagy

The main neuroglia present in the retina are microglia, astrocytes, and Müller cells (Vecino et al., 2016). Of these, microglia play a crucial role in neuroinflammation (Zhang W. et al., 2021), which is a critical process in glaucoma (Guzman-Martinez et al., 2019). Microglia in the retina are resident innate immune cells in neural tissue that perform as neuropathological receptors and are also the primary immune defense against retinal damage (Chen et al., 2022). Under physiological conditions, glial cells secrete neurotrophic factors to promote nerve cell survival and tissue regeneration and perform phagocytosis to eliminate foreign components (Campagno et al., 2021). Microglia activated by stimulation [such as HIOP (Campagno et al., 2021)] secrete inflammatory cytokines to induce neuronal apoptosis, which further contributes to neuroinflammation. Activated microglia can be polarized into either an M1 proinflammatory phenotype or an M2 anti-inflammatory phenotype, and the two can switch between each other in response to different microenvironmental disorders, known as classical activation and alternative activation respectively (Lan et al., 2017). M1 polarized microglia activate the neuroinflammatory response and exacerbate neuronal damage *via* the synthesis and release of injury-mediating cytokines (TNFα, IL-1β, IL-6, IL-12), protein hydrolases (MMPs), and the expression of MHC-II (Ramirez et al., 2017). M2 polarized microglia, secrete anti-inflammatory cytokines and growth factors and play the opposite role of M1 microglia (Lan et al., 2017), promoting phagocytosis and waste removal, neurogenesis, axonal remodeling, or myelin regeneration after injury (Liu et al., 2018). Activated neuroinflammatory pathways may initially restore homeostasis between RGCs and the extracellular environment, with protective effects. However, the overproduction of chemokines, and cytokines and the excessive activation of retina-resident microglia and other innate immune cells, may prompt RGC degeneration (Quaranta et al., 2021). Neuroinflammation may be an important cause of RGC degeneration and targeting M2 microglial polarization could be a potential therapeutic option to treat glaucoma (Cherry et al., 2014; Reboussin et al., 2022).

The proinflammatory cytokine TNF-α produced by M1 microglia was demonstrated to inhibit autophagic flux in neurons and microglia, which further drives M1 polarization in microglia *via* activation of the Akt/mTOR pathway (Jin et al., 2018). Conversely, IL-4, which promotes microglial polarization to the M2 subtype, activates autophagic flux and exerts a protective effect (Tang et al., 2019). Autophagy has been reported to direct microglial activation to the M2 subtype, and consistently, autophagy inhibition promotes polarization toward the proinflammatory M1 subtype (Jin et al., 2018). The M2 subtype is maintained by high autophagic flux, and disruption of autophagic flux results in a shift in the M1-related subtype (Zubova et al., 2022). In conclusion, autophagy degrades proinflammatory proteins, limits the inflammatory process, and exerts a protective effect.

### 3.4 Aging and autophagy

In most tissues, autophagy declines with age, which can lead to the accumulation of certain types of cellular components at potentially toxic levels that can be harmful to cells (Hansen et al., 2018). Dysregulation of autophagy, a cellular catabolic mechanism that removes misfolded proteins, is responsible for a range of neurodegenerative diseases (Lipinski et al., 2010). Autophagy activity is impaired as the brain ages in different species (Hansen et al., 2018). For example, autophagy is reduced in mouse hypothalamic neurons (Kaushik et al., 2012) and the mRNA levels of many ATG are reduced in the aging human brain (Lipinski et al., 2010). Studies on the retina have determined that glaucoma is also a neurodegenerative disease, and the mRNA expression of autophagy regulators (e.g., ATG and Beclin) is reduced (Rodríguez-Muela et al., 2013). In an optic nerve crush model, autophagy-deficiency in aged mice (Ambra1+/gt) increases axonal damage susceptibility (Bell et al., 2020). Autophagy in the retina decreases with age and activation of autophagy shows neuroprotective effects *in vivo* experimental of glaucoma (Bell et al., 2020). Dysregulation of TM autophagy associated with elevated IOP also occurs during aging (Hirt et al., 2018). Overall, there is growing evidence to support the beneficial role of autophagy in the fight against aging and age-related diseases.

### 3.5 Gene mutation and autophagy

OPTN, TANK binding kinase 1 (TBK1), and Myocilin (MYOC) are genes linked to glaucoma (Sears et al., 2019). Mutation in each of these genes may cause this major blinding disease (Sears et al., 2019). E50K and M98K mutants induced excess cell death than wild-type OPTN (Sirohi et al., 2013; Sirohi and Swarup, 2016). E50K mutation causes retinal cell death in the cell culture model and transgenic mouse model by inhibition of autophagy (Chalasani et al., 2014; Zhang S. et al., 2021). M98K is a gain-of-function mutation, overexpression of M98K shows enhanced autophagic signaling leading to endocytic recycling of transferrin receptor (TFRC) degradation and causing cell death (Sirohi et al., 2013). Uncontrolled activation of TBK1 by the mutation that A 2-bp insertion in OPTN leads to impaired autophagy that results in the accumulation of LC3-positive aggregates, which induces ER stress and a retinal cell line, 661 W, cell death (Medchalmi et al., 2021). In addition, duplication of TBK1 can cause glaucoma, for the reason that increased transcription of TBK1 encodes a kinase that phosphorylates OPTN, which recruits LC3B and initiates autophagy (Tucker et al., 2014). The mutant MYOC-induced impaired autophagy leads to TM cell death and HIOP, which is associated with the induction of transcription factor, CCAAT/enhancer-binding protein homologous protein (Stothert et al., 2016; Kasetti et al., 2021; Yan et al., 2022). These evidences indicated that some glaucoma caused by gene mutation is related to impaired autophagy.

## 4 Progress and prospects for research on glaucoma treatment targeting autophagy

Glaucoma is a complex disease with no effective cure. Glaucoma is usually treated with prescription eye drops that function *via* different molecular mechanisms, including prostaglandin analogs (latanoprost, travoprost, bimatoprost, and tafluprost) (Aihara, 2021), β-blockers (Skov et al., 2021), α-adrenergic agonists (Nocentini and Supuran, 2019), cholinergic inhibitors, carbonic anhydrase inhibitors (Jansook et al., 2021) and cholinergic drugs (Faiq et al., 2019). These eye drops reduce IOP by improving the outflow of AH through the trabecular drainage system or by reducing the volume of AH produced by the eye. However, the absorption of eye drop molecules into the bloodstream can result in side effects (Xu et al., 2017). By way of explanation, beta-blockers are connected with cardiac arrhythmia, congestive heart failure, and airway obstruction (Argulian et al., 2019), and carbonic anhydrase inhibitors may provoke thrombocytopenia (Xu et al., 2017). In some patients with advanced glaucoma, eye drops cannot lower the IOP to the required level and surgery or alternative treatment is needed. Surgical treatment to increase AH flow is currently the main method used to reduce IOP. The first laser treatment to enhance the outflow system was performed approximately 50 years ago (Wise and Witter, 1979). At present, the main types of surgery are selective laser trabeculoplasty (Töteberg-Harms and Meier-Gibbons, 2021), glaucoma filtration surgery(Wolters et al., 2021), Ahmed glaucoma valve implantation(a popular glaucoma drainage implant) (Riva et al., 2017), or minimally invasive glaucoma surgery (Kan et al., 2021).

Therapy to lower IOP has proven successful in preserving vision in some glaucoma patients (Heijl et al., 2002; Lichter, 2002), but not all. There is growing evidence that regulation of autophagy to degrade misfolded proteins and remove damaged organelles may be an ideal option for glaucoma treatment (Tsai et al., 2022). However, autophagy can be a "double-edged sword" in various animal models and experimental approaches and requires to be precisely regulated; thus, it is particularly crucial to better perceive the molecular pathways and targeted regulation of autophagy (Park et al., 2018; Ishikawa et al., 2021). Autophagy is a highly conservative, highly dynamic, and complex regulatory process, which can theoretically be stimulated at multiple levels, providing a variety of pharmacological targets for the development of corresponding agonists or antagonists (Zhang Z. et al., 2021). Below, we will discuss the potential mechanisms of various Western and Chinese therapies, emerging biological agents, and stem cell interventions for autophagy in glaucoma treatment as well as their effectiveness in animal models and clinical trials.

### 4.1 Chinese autophagy-targeting therapies for glaucoma

In recent years, Chinese medicine has been effective for treating glaucoma in combination with Western medicine or surgery (Wen-bo et al., 2020; Xirui et al., 2021). In Chinese medicine, it is considered that the pathogenesis of glaucoma is based on “stasis” and “deficiency”, and treatment should be based on activating blood circulation, removing blood stasis, moving qi, and opening depression, which corresponds to the “clearing” effect of autophagy. Autophagy, a current research hotspot, is closely related to glaucomatous optic neuropathy in many ways and has led to initial research progress in using Chinese medicine therapies to regulate autophagy to protect the optic nerve.

In research on Chinese medicine/Chinese herbal extracts, in a rat model of chronic hypertensive glaucoma established by the extrascleral vein cautery (EVC) method, autophagy was significantly activated in retinal cells and the number of autophagic vesicles increased, while chuanxiongzin completely reversed this change in rats by activating the PI3K-Akt/mTOR pathway to inhibit autophagy, acting as a protective agent (Du et al., 2021). The possible signaling pathways through which each herbal medicine/Chinese medicine extract exerts its protective effect by regulating autophagy are shown in Table 2.

**TABLE 2:** Strategies for glaucoma treatment involving autophagy.

Effect	Strategy	Experimental model	Molecular signaling pathway/autophagy markers	Effects of autophagy modulators	Ref
Autophagy induction	The neurosteroid AlloP	To generate an OH model, male SD rats were intracamerally injected with polystyrene microbeads	LC3B-II↑, SQSTM1↓	Prevented apoptosis and protected RGCs with autophagy activation	Ishikawa et al. (2021)
	Rapamycin	Male C57BL/6 J mice were subjected to acute HIOP (90–100 mmHg) for 60 min	mTOR inhibition	Prolonged autophagy activation and improved RGC survival	Russo et al. (2018)
	Tat-beclin 1 peptide or Torin 2	Tg-MYOCY437H mice	Mutant myocilin↓, LC3BII, ATG5, and beclin1↑, p62↓	Tat-beclin 1 peptide and Torin 2 significantly reduced IOP in Tg-MYOCY437H mice	Kasetti et al. (2021)
	Metformin	SD rats of GFS	AMPK/Nrf2 axis ↑, profibrogenic and inflammatory biomarkers↓	Protect against filtrating blebs scar formation in SD rats of GFS.	Li et al. (2020)
	Rapamycin	E50K-OPTN mice	LC3-II, p62↓, TDP-43 aggregation↓	Rapamycin reduce TDP-43 could suppress E50K-mediated apoptosis	Zhang et al. (2021b)
Autophagy inhibition	Blocker of lysosomal degradation (Baf-A1)	For COH, a dose of micromagnetic beads (2 μL) was injected into the anterior chamber of the right eye only once	Autolysosomes↑, p62↑	Biphasic autophagy in RGCs functioned dynamically during cell injury: promoting RGC apoptosis in the early stages (G4d) and resulting in RGC death in the second stage (G3w)	Zhang et al. (2020)
	Rac1 cKO	RGC Rac1 conditional knockout (Rac1 cKO) mice	mTOR↑, Beclin1, LC3, and Bak↓	Activated Rac1 exacerbated RGC injury in COH retinas, and Rac1 cKO significantly reduced apoptotic RGCs	Zhang et al. (2020)
	Overexpression of miR-708 and miR-335-3p	Chronic glaucoma mice were established by laser photocoagulation	ATG3↓, LC3II/I↓, P62↑	Overexpression of miR-708/miR-335-3p alleviated RGC apoptosis	Zhang et al. (2021a)
	Chloroquine	M98K-OPTN mice	TFRC↑	Chloroquine inhibited CASP3 and PARP1 cleavage, increase TFRC.	Sirohi et al. (2013)
	Ligustrazine (TMP)	A rat chronic hypertensive glaucoma model was induced by EVC.	p-PI3K, protein kinase B (p-Akt), and mTOR (p-mTOR)↑	Ligustrazine was neuroprotective in experimental glaucoma by inhibiting autophagy in RGCs	Du et al. (2021)

AlloP, allopregnanolone; SD, Sprague–Dawley; OH: ocular hypertension; LC3, light chain 3 protein; ATG, autophagy-related genes; GFS, glaucoma filtrating surgery; AMPK, AMP-activated protein kinase; Nrf2, nuclear factor erythroid 2-related factor 2; COH, chronic ocular hypertension; Rac1 cKO, Rac1 conditional knockout; OPTN, optineurin; PI3K, phosphatidylinositol 3-kinase; TFRC, transferrin receptor; EVC, episcleral vein cauterization; p-PI3K, phosphorylated.

Clinical data show that acupuncture combined with conventional Western medicine can effectively reduce intraocular pressure and significantly improve the degree of optic nerve atrophy in patients with glaucoma (Wang, 2017). Acupuncture also reduces visual field defects and improves ocular artery hemodynamics and visual acuity (Leszczynska et al., 2018; Lan and Xiaojie, 2020). At present, studies involving the optic neuroprotective effects of acupuncture therapy have been limited to clinical data analysis, but in the neuroprotective effect of cranial nerves, acupuncture can play a protective role by inhibiting autophagy through the mTORC1–ULK complex–Beclin1pathway (Liu et al., 2016).

### 4.2 Western autophagy-targeted medical therapy for glaucoma

Enhanced autophagy efficacy may reduce the number of toxic protein aggregates, provide an effective stress response, and prevent or reduce cell death by degrading inessential components for energy and supporting adaptive protein synthesis. Allogeneic progesterone (Allop) increases autophagy vesicles, activates autophagy, and protects RGCs *via* GABR/GABAA receptors (Ishikawa et al., 2021). Rapamycin inhibits mTOR and therefore induces autophagy in peripheral organs and the CNS. Using an optic nerve transection model, rapamycin-treated mice showed a 40% improvement in RGC survival 10 days after axotomy, exerting a beneficial effect (Rodríguez-Muela et al., 2012). Rapamycin can also increase the RGC number and visual function, and reduce apoptosis of E50K-OPTN mice (Shen et al., 2011; Zhang S. et al., 2021). Stimulation of autophagy *via* tat-beclin 1 peptide or Torin 2 rescued MYOC-associated transgenic mice model (Kasetti et al., 2021). In various *in vivo* experimental models of glaucoma, the induction of autophagy by pharmacological or genetic manipulation can improve RGC survival (Table 2).

Evidence suggests that autophagy induction has a neuroprotective effect on glaucoma-damaged RGCs; however, autophagy induction cannot be directly said to be a panacea for neurodegenerative disease, and the induction of autophagosome biogenesis may be harmful if autophagosome accumulation is not effectively cleared. It has been shown that autophagy inhibition also has neuroprotective effect on glaucoma-damaged RGCs. In a rat model of chronic HIOP glaucoma, autophagy was significantly activated in RGCs (Deng et al., 2013). The number of TUNEL-positive cells was reduced in the GCL since autophagy inhibition by the autophagy inhibitor 3-Methyladenine (3-MA), indicating that apoptosis occurred in RGCs after autophagy-induced chronic IOP elevation (Park et al., 2012). M98K-OPTN potentiates the degradation of TFRC by autophagy and contributes to the death of RGCs; chloroquine (CQ) or the knockdown of Atg5 by shRNA significantly reduced cell death mediated by M98K (Sirohi et al., 2013; Swarup and Sayyad, 2018). The possible signaling pathways through which each Western drug exerts its protective effects by modulating autophagy are shown in Table 2.

Currently the role of autophagy in glaucoma is still controversial; it relies on the type of glaucoma model, the target of pharmacological treatment, and the period of drug administration (Wang et al., 2015; Russo et al., 2018). Studies have shown that is chemic/reperfusion can trigger autophagy in the first few hours; although RGC loss at this time, autophagy as the mechanism of self-adaptation under stress conditions might maintain cellular homeostasis and reduce the harm (Russo et al., 2018). The subsequent protective effect of rapamycin also confirmed this point (Russo et al., 2018). However, it is demonstrated that rapamycin aggravated RGC loss and axon demyelination at day 14 of reperfusion, as mTOR activity plays a crucial role in determining neuronal survival (Chen et al., 2016). The role of autophagy in glaucoma is multifunctional and dynamic. Consequently, a better comprehension of the underlying mechanisms of autophagy dysfunction in glaucoma is essential for developing therapeutic approaches.

### 4.3 Emerging therapies (biologics/stem cells) and autophagy

RGCs degenerate in glaucoma, resulting in permanent vision loss. Cell replacement strategies are considered potential therapies for RGC loss. Researchers have recently explored a potential replacement therapy based on human stem cells that can differentiate into RGC cells (Nuzzi and Tridico, 2017; Chang et al., 2019). However, scaling up donor cells, advancing cell differentiation, and promoting cell durability remain challenges in replacement therapies.

Elaborate mechanisms render mesenchymal stem cells (MSCs) neuroprotective, including modulating neuroinflammation, enhancing cell survival, increasing neurogenesis, and regulating ubiquitinated proteins (Shin et al., 2014). It has been documented that hUC-MSCs have a protective effect on retinal neurons (Wang et al., 2021), and MSC-EVs also exert a neuroprotective effect on the retina by reducing neuroinflammation and neuronal apoptosis (Mathew et al., 2019). At present, studies involving the optic neuroprotective effects of MSCs and MSC-EVs are more limited to phenotypic analysis, and in the brain, MSCs can improve neuronal survival by activating autophagy (Shin et al., 2014; Ceccariglia et al., 2020).

## 5 Conclusion and future perspectives

In summary, autophagy is an important metabolic process that maintains cellular homeostasis and autophagy dysfunction influences in the pathogenesis of glaucoma. Although the relationship between autophagy and glaucoma remains controversial and it is uncertain whether autophagy dysfunction plays a determinant role in disease progression, increasing evidence suggests that autophagy induction has a neuroprotective effect in glaucoma (Russo et al., 2018). Overactivated autophagy may also cause the development of autophagic cell death (Shi et al., 2012). In any case, since autophagy has a waste-disposal function, its activation and inhibition may be a novel therapeutic strategy in the context of glaucoma.

It has been shown that autophagy activity may not be simply positive or negative for cell survival, but that its effects depend on the timing, amplitude and target of autophagy activation (Ferrucci et al., 2018). Thus, more detailed studies are required to determine the timing and dosage of autophagy modulators that moderately activate autophagy and maintain it at relatively normal metabolic levels. It has been found that the induction of acute autophagy in a retinal ischemia-reperfusion model continues for the first few hours and then declines (Russo et al., 2018; Tang et al., 2021). While the activation of autophagy has been mentioned as having a protective effect, inhibition of autophagy has also exhibited a protective effect. The initial increase in autophagy may be compensatory effect, while activating autophagy with drugs can only have protective effect after autophagy subsequently decreases. Thus, it is necessary to perform a precise assessment of autophagy at various time points in the disease model and make minor adjustments to drug concentrations to characterize the potential time-dependent changes in autophagy and the optimal concentration of drugs for obtaining accurate data on the protective effects of modulating autophagy.

There have been many studies on the role of autophagy in the pathogenesis and treatment of glaucoma in the experiment. HIOP, oxidative stress, neuroinflammation, aging and gene mutation may all lead to the dysregulation of autophagy, and regulating autophagy is also a target for the treatment of glaucoma. However, progress in evaluating the characteristics of autophagy in the pathogenesis of human glaucoma and therapeutic options depend heavily on the advancement of methods to monitor autophagy activity in humans. For retina, it is not as easy to obtain human fresh biopsies as blood, muscle, and adipose. Autopsy materials are heterogeneity and can only be measured statically (Klionsky et al., 2021). Monitoring autophagy dynamic activity in glaucoma and truly autophagy fluxes of some human tissues remain relatively challenging. For example, it is not rigorous to simply assay levels of endogenous LC3-II in retinal tissue samples and use this as a proxy for autophagy flux. Because there is no real effective degradation of LC3II, if there is an impaired fusion of autophagosomes with lysosomes or impaired lysosomal activity late in the autophagy pathway, an increase in LC3II does not represent increased autophagy activity (Mizushima et al., 2010). There are studies suggest that the total cellular expression level of p62 is generally considered inversely proportional to autophagy activity (Klionsky et al., 2021), but it is unclear whether p62 is degraded only or partially by the ubiquitin-proteasome pathway of autophagy (Mizushima et al., 2010). This also means that most experts in the field do not consider some of the methods used in the literature to be appropriate measures of autophagic flux (or autophagic activity). It is essential to develop real-time monitoring methods for autophagy and measurement of biomarkers that can be applied in clinical practice.

Autophagy-oriented strategies may offer a new and promising alternative for the development of drugs for glaucoma. In addition, it would be interesting to investigate emerging targeted delivery molecules to modulate autophagy and reduce side effects, and to investigate the use of biological agents such as stem cell extracellular vesicles or engineered exosomes to modulate autophagy and achieve a protective effect consistent with that of stem cells.

## Abbreviations

RGCs, retinal ganglion cells; MSVI, moderate and severe vision impairment; CNS, central nervous system; IOP, intraocular pressure; PD, Parkinson’s disease; HIOP, high IOP; TM, trabecular meshwork; LC3, light chain 3 protein; ATG, autophagy related genes; PI3K, phosphatidylinositol 3-kinase; PI3P, phosphatidylinositol 3-phosphate; ATG5-12/16L1, ATG5-ATG12 conjugate binds to ATG16L1 to generate the autophagy elongation complex; PE, phosphatidylethanolamine; TFEB, transcription factor EB; OPTN, optineurin; AH, aqueous humor; SC, Schlemm’s canal; PC, primary cilia; ROS, reactive oxygen species; NF-κB, nuclear factor kappa-B; IL-1α, interleukin-1α; ELAM-1, endothelial leukocyte adhesion molecule-1; TBK1, TANK binding kinase 1, MYOC, Myocilin; TFRC, transferrin receptor; EVC, extra-scleral vein cautery; Allop, Allogeneic progesterone; 3-MA, 3-Methyladenine; CQ, chloroquine; MSCs, Mesenchymal stem cells.

## References

[B1] Acott T. S. Kelley M. J. Keller K. E. Vranka J. A. Abu-Hassan D. W. Li X. (2014). Intraocular pressure homeostasis: Maintaining balance in a high-pressure environment. J. Ocul. Pharmacol. Ther. 30 (2-3), 94–101. 10.1089/jop.2013.0185 24401029 PMC3991985

[B2] Aihara M. (2021). Prostanoid receptor agonists for glaucoma treatment. Jpn. J. Ophthalmol. 65 (5), 581–590. 10.1007/s10384-021-00844-6 34228229

[B3] Argulian E. Bangalore S. Messerli F. H. (2019). Misconceptions and facts about beta-blockers. Am. J. Med. 132 (7), 816–819. 10.1016/j.amjmed.2019.01.039 30817899

[B4] Baetz N. W. Hoffman E. A. Yool A. J. Stamer W. D. (2009). Role of aquaporin-1 in trabecular meshwork cell homeostasis during mechanical strain. Exp. Eye Res. 89 (1), 95–100. 10.1016/j.exer.2009.02.018 19268465 PMC2733866

[B5] Bell K. Rosignol I. Sierra-Filardi E. Rodriguez-Muela N. Schmelter C. Cecconi F. (2020). Age related retinal Ganglion cell susceptibility in context of autophagy deficiency. Cell Death Discov. 6, 21. 10.1038/s41420-020-0257-4 32337073 PMC7165178

[B6] Benyair R. Eisenberg-Lerner A. Merbl Y. (2022). Maintaining Golgi homeostasis: A balancing act of two proteolytic pathways. Cells 11 (5), 780. 10.3390/cells11050780 35269404 PMC8909885

[B7] Bonomi L. Marchini G. Marraffa M. Bernardi P. De Franco I. Perfetti S. (1998). Prevalence of glaucoma and intraocular pressure distribution in a defined population. The Egna-Neumarkt Study. Ophthalmology 105 (2), 209–215. 10.1016/s0161-6420(98)92665-3 9479277

[B8] Borrás T. (2003). Gene expression in the trabecular meshwork and the influence of intraocular pressure. Prog. Retin. Eye Res. 22 (4), 435–463. 10.1016/s1350-9462(03)00018-1 12742391

[B9] Boya P. (2017). Why autophagy is good for retinal ganglion cells? Eye (Lond) 31 (2), 185–190. 10.1038/eye.2016.278 27983732 PMC5306464

[B10] Bradley J. M. Kelley M. J. Zhu X. Anderssohn A. M. Alexander J. P. Acott T. S. (2001). Effects of mechanical stretching on trabecular matrix metalloproteinases. Invest. Ophthalmol. Vis. Sci. 42 (7), 1505–1513. 11381054

[B11] Bressan C. Saghatelyan A. Czyzewska M. M. Brodzki M. Mozrzymas J. W. (2020). GABAA receptor β2E155 residue located at the agonist-binding site is involved in the receptor gating. Front. Cell. Neurosci. 14, 2. 10.3389/fncel.2020.00002 32116555 PMC7026498

[B12] Campagno K. E. Lu W. Jassim A. H. Albalawi F. Cenaj A. Tso H. Y. (2021). Rapid morphologic changes to microglial cells and upregulation of mixed microglial activation state markers induced by P2X7 receptor stimulation and increased intraocular pressure. J. Neuroinflammation 18 (1), 217. 10.1186/s12974-021-02251-7 34544431 PMC8454080

[B13] Carreon T. van der Merwe E. Fellman R. L. Johnstone M. Bhattacharya S. K. (2017). Aqueous outflow - a continuum from trabecular meshwork to episcleral veins. Prog. Retin. Eye Res. 57, 108–133. 10.1016/j.preteyeres.2016.12.004 28028002 PMC5350024

[B14] Ceccariglia S. Cargnoni A. Silini A. R. Parolini O. (2020). Autophagy: A potential key contributor to the therapeutic action of mesenchymal stem cells. Autophagy 16 (1), 28–37. 10.1080/15548627.2019.1630223 31185790 PMC6984485

[B15] Chalasani M. L. Kumari A. Radha V. Swarup G. (2014). E50K-OPTN-induced retinal cell death involves the Rab GTPase-activating protein, TBC1D17 mediated block in autophagy. PLoS One 9 (4), e95758. 10.1371/journal.pone.0095758 24752605 PMC3994150

[B16] Chang E. E. Goldberg J. L. (2012). Glaucoma 2.0: Neuroprotection, neuroregeneration, neuroenhancement. Ophthalmology 119 (5), 979–986. 10.1016/j.ophtha.2011.11.003 22349567 PMC3343191

[B17] Chang K. C. Liu P. F. Chang C. H. Lin Y. C. Chen Y. J. Shu C. W. (2022). The interplay of autophagy and oxidative stress in the pathogenesis and therapy of retinal degenerative diseases. Cell Biosci. 12 (1), 1. 10.1186/s13578-021-00736-9 34980273 PMC8725349

[B18] Chang K. C. Sun C. Cameron E. G. Madaan A. Wu S. Xia X. (2019). Opposing effects of growth and differentiation factors in cell-fate specification. Curr. Biol. 29 (12), 1963–1975. 10.1016/j.cub.2019.05.011 31155355 PMC6581615

[B19] Chen D. Peng C. Ding X. M. Wu Y. Zeng C. J. Xu L. (2022). Interleukin-4 promotes microglial polarization toward a neuroprotective phenotype after retinal ischemia/reperfusion injury. Neural Regen. Res. 17 (12), 2755–2760. 10.4103/1673-5374.339500 35662225 PMC9165374

[B20] Chen G. Tang L. Wei W. Li Z. Li Y. Duan X. (2016). mTOR regulates neuroprotective effect of immunized CD4+Foxp3+ T cells in optic nerve ischemia. Sci. Rep. 6, 37805. 10.1038/srep37805 27886260 PMC5122903

[B21] Chen Q. Xi X. Zeng Y. He Z. Zhao J. Li Y. (2019). Acteoside inhibits autophagic apoptosis of retinal ganglion cells to rescue glaucoma-induced optic atrophy. J. Cell. Biochem. 120 (8), 13133–13140. 10.1002/jcb.28586 31021425 PMC6618276

[B22] Cherry J. D. Olschowka J. A. O'Banion M. K. (2014). Neuroinflammation and M2 microglia: The good, the bad, and the inflamed. J. Neuroinflammation 11, 98. 10.1186/1742-2094-11-98 24889886 PMC4060849

[B23] Conway O. Akpinar H. A. Rogov V. V. Kirkin V. (2020). Selective autophagy receptors in neuronal Health and disease. J. Mol. Biol. 432 (8), 2483–2509. 10.1016/j.jmb.2019.10.013 31654670

[B24] Deng S. Wang M. Yan Z. Tian Z. Chen H. Yang X. (2013). Autophagy in retinal ganglion cells in a rhesus monkey chronic hypertensive glaucoma model. PLoS One 8 (10), e77100. 10.1371/journal.pone.0077100 24143204 PMC3797129

[B25] Du H. Y. Wang R. Li J. L. Luo H. Xie X. Y. Yan R. (2021). Ligustrazine protects against chronic hypertensive glaucoma in rats by inhibiting autophagy via the PI3K-Akt/mTOR pathway. Mol. Vis. 27, 725–733. 35035207 PMC8711580

[B26] Ebrahimi-Fakhari D. Wahlster L. McLean P. J. (2012). Protein degradation pathways in Parkinson's disease: Curse or blessing. Acta Neuropathol. 124 (2), 153–172. 10.1007/s00401-012-1004-6 22744791 PMC3417142

[B28] Faiq M. A. Wollstein G. Schuman J. S. Chan K. C. (2019). Cholinergic nervous system and glaucoma: From basic science to clinical applications. Prog. Retin. Eye Res. 72, 100767. 10.1016/j.preteyeres.2019.06.003 31242454 PMC6739176

[B29] Feng Y. Yao Z. Klionsky D. J. (2015). How to control self-digestion: Transcriptional, post-transcriptional, and post-translational regulation of autophagy. Trends Cell Biol. 25 (6), 354–363. 10.1016/j.tcb.2015.02.002 25759175 PMC4441840

[B30] Fernández-Durango R. Fernández-Martínez A. García-Feijoo J. Castillo A. de la Casa J. M. García-Bueno B. (2008). Expression of nitrotyrosine and oxidative consequences in the trabecular meshwork of patients with primary open-angle glaucoma. Invest. Ophthalmol. Vis. Sci. 49 (6), 2506–2511. 10.1167/iovs.07-1363 18296660

[B31] Ferrucci M. Biagioni F. Ryskalin L. Limanaqi F. Gambardella S. Frati A. (2018). Ambiguous effects of autophagy activation following hypoperfusion/ischemia. Int. J. Mol. Sci. 19 (9), 2756. 10.3390/ijms19092756 30217100 PMC6163197

[B32] Frake R. A. Ricketts T. Menzies F. M. Rubinsztein D. C. (2015). Autophagy and neurodegeneration. J. Clin. Invest. 125 (1), 65–74. 10.1172/jci73944 25654552 PMC4382230

[B33] Galluzzi L. Baehrecke E. H. Ballabio A. Boya P. Bravo-San Pedro J. M. Cecconi F. (2017). Molecular definitions of autophagy and related processes. Embo J. 36 (13), 1811–1836. 10.15252/embj.201796697 28596378 PMC5494474

[B34] GBD 2019 Blindness and Vision Impairment Collaborators; Vision Loss Expert Group of the Global Burden of Disease Study (2021). Causes of blindness and vision impairment in 2020 and trends over 30 years, and prevalence of avoidable blindness in relation to VISION 2020: The right to sight: An analysis for the global burden of disease study. Lancet. Glob. Health 9 (2), e144–e160. 10.1016/s2214-109x(20)30489-7 33275949 PMC7820391

[B35] Glick D. Barth S. Macleod K. F. (2010). Autophagy: Cellular and molecular mechanisms. J. Pathol. 221 (1), 3–12. 10.1002/path.2697 20225336 PMC2990190

[B36] Gupta N. Yücel Y. H. (2007). Glaucoma as a neurodegenerative disease. Curr. Opin. Ophthalmol. 18 (2), 110–114. 10.1097/ICU.0b013e3280895aea 17301611

[B37] Guzman-Martinez L. Maccioni R. B. Andrade V. Navarrete L. P. Pastor M. G. Ramos-Escobar N. (2019). Neuroinflammation as a common feature of neurodegenerative disorders. Front. Pharmacol. 10, 1008. 10.3389/fphar.2019.01008 31572186 PMC6751310

[B38] Hansen M. Rubinsztein D. C. Walker D. W. (2018). Autophagy as a promoter of longevity: Insights from model organisms. Nat. Rev. Mol. Cell Biol. 19 (9), 579–593. 10.1038/s41580-018-0033-y 30006559 PMC6424591

[B39] Harvey A. R. Ooi J. W. Rodger J. (2012). Neurotrophic factors and the regeneration of adult retinal ganglion cell axons. Int. Rev. Neurobiol. 106, 1–33. 10.1016/b978-0-12-407178-0.00002-8 23211458

[B40] He J. Deng L. Liu H. Chen T. Chen S. Xia S. (2019). BCL2L10/BECN1 modulates hepatoma cells autophagy by regulating PI3K/AKT signaling pathway. Aging (Albany NY) 11 (2), 350–370. 10.18632/aging.101737 30696802 PMC6366968

[B41] Heijl A. Leske M. C. Bengtsson B. Hyman L. Bengtsson B. Hussein M. (2002). Reduction of intraocular pressure and glaucoma progression: Results from the early manifest glaucoma trial. Arch. Ophthalmol. 120 (10), 1268–1279. 10.1001/archopht.120.10.1268 12365904

[B42] Hirt J. Liton P. B. (2017). Autophagy and mechanotransduction in outflow pathway cells. Exp. Eye Res. 158, 146–153. 10.1016/j.exer.2016.06.021 27373974 PMC5199638

[B43] Hirt J. Porter K. Dixon A. McKinnon S. Liton P. B. (2018). Contribution of autophagy to ocular hypertension and neurodegeneration in the DBA/2J spontaneous glaucoma mouse model. Cell Death Discov. 4, 14. 10.1038/s41420-018-0077-y PMC6127277 30210817

[B44] Ishikawa M. Takaseki S. Yoshitomi T. Covey D. F. Zorumski C. F. Izumi Y. (2021). The neurosteroid allopregnanolone protects retinal neurons by effects on autophagy and GABRs/GABA(A) receptors in rat glaucoma models. Autophagy 17 (3), 743–760. 10.1080/15548627.2020.1731270 32070183 PMC8032250

[B45] Ito Y. A. Di Polo A. (2017). Mitochondrial dynamics, transport, and quality control: A bottleneck for retinal ganglion cell viability in optic neuropathies. Mitochondrion 36, 186–192. 10.1016/j.mito.2017.08.014 28866056

[B46] Jansook P. Hnin H. M. Loftsson T. Stefánsson E. (2021). Cyclodextrin-based formulation of carbonic anhydrase inhibitors for ocular delivery - a review. Int. J. Pharm. 606, 120955. 10.1016/j.ijpharm.2021.120955 34332063

[B47] Jin M. M. Wang F. Qi D. Liu W. W. Gu C. Mao C. J. (2018). A critical role of autophagy in regulating microglia polarization in neurodegeneration. Front. Aging Neurosci. 10, 378. 10.3389/fnagi.2018.00378 30515090 PMC6256089

[B48] Johansen T. Lamark T. (2020). Selective autophagy: ATG8 family proteins, LIR motifs and cargo receptors. J. Mol. Biol. 432 (1), 80–103. 10.1016/j.jmb.2019.07.016 31310766

[B49] Kan J. T. Betzler B. K. Lim S. Y. Ang B. C. H. (2021). Anterior segment imaging in minimally invasive glaucoma surgery - a systematic review. Acta Ophthalmol. 100, e617–e634. 10.1111/aos.14962 34250742

[B50] Kasetti R. B. Maddineni P. Kiehlbauch C. Patil S. Searby C. C. Levine B. (2021). Autophagy stimulation reduces ocular hypertension in a murine glaucoma model via autophagic degradation of mutant myocilin. JCI Insight 6 (5), e143359. 10.1172/jci.insight.143359 33539326 PMC8021112

[B51] Kaushik S. Arias E. Kwon H. Lopez N. M. Athonvarangkul D. Sahu S. (2012). Loss of autophagy in hypothalamic POMC neurons impairs lipolysis. EMBO Rep. 13 (3), 258–265. 10.1038/embor.2011.260 22249165 PMC3323137

[B52] Kaushik S. Cuervo A. M. (2018). The coming of age of chaperone-mediated autophagy. Nat. Rev. Mol. Cell Biol. 19 (6), 365–381. 10.1038/s41580-018-0001-6 29626215 PMC6399518

[B53] Kim J. Kundu M. Viollet B. Guan K. L. (2011). AMPK and mTOR regulate autophagy through direct phosphorylation of Ulk1. Nat. Cell Biol. 13 (2), 132–141. 10.1038/ncb2152 21258367 PMC3987946

[B54] Kimura A. Namekata K. Guo X. Noro T. Harada C. Harada T. (2017). Targeting oxidative stress for treatment of glaucoma and optic neuritis. Oxid. Med. Cell. Longev. 2017, 2817252. 10.1155/2017/2817252 28270908 PMC5320364

[B55] Kiriyama Y. Nochi H. (2015). The function of autophagy in neurodegenerative diseases. Int. J. Mol. Sci. 16 (11), 26797–26812. 10.3390/ijms161125990 26569220 PMC4661849

[B56] Klionsky D. J. Abdel-Aziz A. K. Abdelfatah S. Abdellatif M. Abdoli A. Abel S. (2021). Guidelines for the use and interpretation of assays for monitoring autophagy (4th edition)^1^ . Autophagy 17 (1), 1–382. 10.1080/15548627.2020.1797280 33634751 PMC7996087

[B57] Ko F. Boland M. V. Gupta P. Gadkaree S. K. Vitale S. Guallar E. (2016). Diabetes, triglyceride levels, and other risk factors for glaucoma in the national Health and nutrition examination survey 2005-2008. Invest. Ophthalmol. Vis. Sci. 57 (4), 2152–2157. 10.1167/iovs.15-18373 27111561 PMC4849858

[B58] Kraft C. Peter M. Hofmann K. (2010). Selective autophagy: Ubiquitin-mediated recognition and beyond. Nat. Cell Biol. 12 (9), 836–841. 10.1038/ncb0910-836 20811356

[B59] Kruszewski M. (2003). Labile iron pool: The main determinant of cellular response to oxidative stress. Mutat. Res. 531 (1-2), 81–92. 10.1016/j.mrfmmm.2003.08.004 14637247

[B60] Kuma A. Hatano M. Matsui M. Yamamoto A. Nakaya H. Yoshimori T. (2004). The role of autophagy during the early neonatal starvation period. Nature 432 (7020), 1032–1036. 10.1038/nature03029 15525940

[B61] Lan J. Xiaojie M. (2020). Effect of acupuncture on vision recovery and ocular hemodynamics in patients with glaucomatous optic atrophy. J Mod. J. Integr. Traditional Chin. West. Med. 29 (27), 3003–3007.

[B62] Lan X. Han X. Li Q. Li Q. Gao Y. Cheng T. (2017). Pinocembrin protects hemorrhagic brain primarily by inhibiting toll-like receptor 4 and reducing M1 phenotype microglia. Brain Behav. Immun. 61, 326–339. 10.1016/j.bbi.2016.12.012 28007523 PMC5453178

[B63] Leszczynska A. Ramm L. Spoerl E. Pillunat L. E. Terai N. (2018). The short-term effect of acupuncture on different ocular blood flow parameters in patients with primary open-angle glaucoma: A randomized, clinical study. Clin. Ophthalmol. 12, 1285–1291. 10.2147/opth.S170396 30050281 PMC6055908

[B64] Li G. Luna C. Liton P. B. Navarro I. Epstein D. L. Gonzalez P. (2007). Sustained stress response after oxidative stress in trabecular meshwork cells. Mol. Vis. 13, 2282–2288. 18199969 PMC3158032

[B65] Li L. Tan J. Miao Y. Lei P. Zhang Q. (2015). ROS and autophagy: Interactions and molecular regulatory mechanisms. Cell. Mol. Neurobiol. 35 (5), 615–621. 10.1007/s10571-015-0166-x 25722131 PMC11486209

[B66] Li X. Leng Y. Jiang Q. Wang Z. Luo P. Zhang C. (2020). Eye drops of metformin prevents fibrosis after glaucoma filtration surgery in rats via activating AMPK/Nrf2 signaling pathway. Front. Pharmacol. 11, 1038. 10.3389/fphar.2020.01038 32903813 PMC7438907

[B67] Lichter P. R. (2002). Impact of intraocular pressure reduction on glaucoma progression. Jama 288 (20), 2607–2608. 10.1001/jama.288.20.2607 12444875

[B68] Lipinski M. M. Zheng B. Lu T. Yan Z. Py B. F. Ng A. (2010). Genome-wide analysis reveals mechanisms modulating autophagy in normal brain aging and in Alzheimer's disease. Proc. Natl. Acad. Sci. U. S. A. 107 (32), 14164–14169. 10.1073/pnas.1009485107 20660724 PMC2922576

[B69] Liton P. B. (2016). The autophagic lysosomal system in outflow pathway physiology and pathophysiology. Exp. Eye Res. 144, 29–37. 10.1016/j.exer.2015.07.013 26226231 PMC4698029

[B70] Liu W. Shang G. Yang S. Huang J. Xue X. Lin Y. (2016). Electroacupuncture protects against ischemic stroke by reducing autophagosome formation and inhibiting autophagy through the mTORC1-ULK1 complex-Beclin1 pathway. Int. J. Mol. Med. 37 (2), 309–318. 10.3892/ijmm.2015.2425 26647915 PMC4716798

[B71] Liu X. Wen S. Yan F. Liu K. Liu L. Wang L. (2018). Salidroside provides neuroprotection by modulating microglial polarization after cerebral ischemia. J. Neuroinflammation 15 (1), 39. 10.1186/s12974-018-1081-0 29426336 PMC5807735

[B72] Madhu L. N. Kodali M. Shetty A. K. (2022). Promise of metformin for preventing age-related cognitive dysfunction. Neural Regen. Res. 17 (3), 503–507. 10.4103/1673-5374.320971 34380878 PMC8504370

[B73] Mathew B. Ravindran S. Liu X. Torres L. Chennakesavalu M. Huang C. C. (2019). Mesenchymal stem cell-derived extracellular vesicles and retinal ischemia-reperfusion. Biomaterials 197, 146–160. 10.1016/j.biomaterials.2019.01.016 30654160 PMC6425741

[B74] Mattson M. P. Gleichmann M. Cheng A. (2008). Mitochondria in neuroplasticity and neurological disorders. Neuron 60 (5), 748–766. 10.1016/j.neuron.2008.10.010 19081372 PMC2692277

[B75] Medchalmi S. Tare P. Sayyad Z. Swarup G. (2021). A glaucoma- and ALS-associated mutant of OPTN induces neuronal cell death dependent on Tbk1 activity, autophagy and ER stress. Febs J. 288 (15), 4576–4595. 10.1111/febs.15752 33548116

[B76] Menzies F. M. Fleming A. Caricasole A. Bento C. F. Andrews S. P. Ashkenazi A. (2017). Autophagy and neurodegeneration: Pathogenic mechanisms and therapeutic opportunities. Neuron 93 (5), 1015–1034. 10.1016/j.neuron.2017.01.022 28279350

[B77] Metaxakis A. Ploumi C. Tavernarakis N. (2018). Autophagy in age-associated neurodegeneration. Cells 7 (5), 37. 10.3390/cells7050037 29734735 PMC5981261

[B78] Mijaljica D. Nazarko T. Y. Brumell J. H. Huang W. P. Komatsu M. Prescott M. (2012). Receptor protein complexes are in control of autophagy. Autophagy 8 (11), 1701–1705. 10.4161/auto.21332 22874568 PMC3494607

[B79] Mizushima N. Levine B. (2020). Autophagy in human diseases. N. Engl. J. Med. 383 (16), 1564–1576. 10.1056/NEJMra2022774 33053285

[B80] Mizushima N. (2020). The ATG conjugation systems in autophagy. Curr. Opin. Cell Biol. 63, 1–10. 10.1016/j.ceb.2019.12.001 31901645

[B81] Mizushima N. Yoshimori T. Levine B. (2010). Methods in mammalian autophagy research. Cell 140 (3), 313–326. 10.1016/j.cell.2010.01.028 20144757 PMC2852113

[B82] Nettesheim A. Shim M. S. Dixon A. Raychaudhuri U. Gong H. Liton P. B. (2020). Cathepsin B localizes in the caveolae and participates in the proteolytic cascade in trabecular meshwork cells. Potential new drug target for the treatment of glaucoma. J. Clin. Med. 10 (1), 78. 10.3390/jcm10010078 33379277 PMC7795952

[B83] Nixon R. A. Wegiel J. Kumar A. Yu W. H. Peterhoff C. Cataldo A. (2005). Extensive involvement of autophagy in alzheimer disease: An immuno-electron microscopy study. J. Neuropathol. Exp. Neurol. 64 (2), 113–122. 10.1093/jnen/64.2.113 15751225

[B84] Nocentini A. Supuran C. T. (2019). Adrenergic agonists and antagonists as antiglaucoma agents: A literature and patent review (2013-2019). Expert Opin. Ther. Pat. 29 (10), 805–815. 10.1080/13543776.2019.1665023 31486689

[B85] Nthiga T. M. Shrestha B. K. Bruun J. A. Larsen K. B. Lamark T. Johansen T. (2021). Regulation of Golgi turnover by CALCOCO1-mediated selective autophagy. J. Cell Biol. 220 (6), e202006128. 10.1083/jcb.202006128 33871553 PMC8059076

[B86] Nuzzi R. Tridico F. (2017). Glaucoma: Biological trabecular and neuroretinal pathology with perspectives of therapy innovation and preventive diagnosis. Front. Neurosci. 11, 494. 10.3389/fnins.2017.00494 28928631 PMC5591842

[B87] Pan T. Kondo S. Le W. Jankovic J. (2008). The role of autophagy-lysosome pathway in neurodegeneration associated with Parkinson's disease. Brain 131 (8), 1969–1978. 10.1093/brain/awm318 18187492

[B88] Park H. L. Kim J. H. Park C. K. (2018). Different contributions of autophagy to retinal ganglion cell death in the diabetic and glaucomatous retinas. Sci. Rep. 8 (1), 13321. 10.1038/s41598-018-30165-7 30190527 PMC6127281

[B89] Park H. Y. Kim J. H. Park C. K. (2012). Activation of autophagy induces retinal ganglion cell death in a chronic hypertensive glaucoma model. Cell Death Dis. 3 (4), e290. 10.1038/cddis.2012.26 22476098 PMC3358006

[B90] Parzych K. R. Klionsky D. J. (2014). An overview of autophagy: Morphology, mechanism, and regulation. Antioxid. Redox Signal. 20 (3), 460–473. 10.1089/ars.2013.5371 23725295 PMC3894687

[B91] Piras A. Gianetto D. Conte D. Bosone A. Vercelli A. (2011). Activation of autophagy in a rat model of retinal ischemia following high intraocular pressure. PLoS One 6 (7), e22514. 10.1371/journal.pone.0022514 21799881 PMC3142183

[B92] Porter K. M. Jeyabalan N. Liton P. B. (2014). MTOR-independent induction of autophagy in trabecular meshwork cells subjected to biaxial stretch. Biochim. Biophys. Acta 1843 (6), 1054–1062. 10.1016/j.bbamcr.2014.02.010 24583119 PMC3988584

[B93] Puertollano R. Ferguson S. M. Brugarolas J. Ballabio A. (2018). The complex relationship between TFEB transcription factor phosphorylation and subcellular localization. Embo J. 37 (11), e98804. 10.15252/embj.201798804 29764979 PMC5983138

[B94] Quaranta L. Bruttini C. Micheletti E. Konstas A. G. P. Michelessi M. Oddone F. (2021). Glaucoma and neuroinflammation: An overview. Surv. Ophthalmol. 66 (5), 693–713. 10.1016/j.survophthal.2021.02.003 33582161

[B95] Ramirez A. I. de Hoz R. Salobrar-Garcia E. Salazar J. J. Rojas B. Ajoy D. (2017). The role of microglia in retinal neurodegeneration: Alzheimer's disease, Parkinson, and glaucoma. Front. Aging Neurosci. 9, 214. 10.3389/fnagi.2017.00214 28729832 PMC5498525

[B96] Ravikumar B. Vacher C. Berger Z. Davies J. E. Luo S. Oroz L. G. (2004). Inhibition of mTOR induces autophagy and reduces toxicity of polyglutamine expansions in fly and mouse models of Huntington disease. Nat. Genet. 36 (6), 585–595. 10.1038/ng1362 15146184

[B97] Reboussin É. Buffault J. Brignole-Baudouin F. Réaux-Le Goazigo A. Riancho L. Olmiere C. (2022). Evaluation of neuroprotective and immunomodulatory properties of mesenchymal stem cells in an *ex vivo* retinal explant model. J. Neuroinflammation 19 (1), 63. 10.1186/s12974-022-02418-w 35236378 PMC8892697

[B98] Riva I. Roberti G. Katsanos A. Oddone F. Quaranta L. (2017). A review of the ahmed glaucoma valve implant and comparison with other surgical operations. Adv. Ther. 34 (4), 834–847. 10.1007/s12325-017-0503-1 28283892

[B99] Rodríguez-Muela N. Germain F. Mariño G. Fitze P. S. Boya P. (2012). Autophagy promotes survival of retinal ganglion cells after optic nerve axotomy in mice. Cell Death Differ. 19 (1), 162–169. 10.1038/cdd.2011.88 21701497 PMC3252838

[B100] Rodríguez-Muela N. Koga H. García-Ledo L. de la Villa P. de la Rosa E. J. Cuervo A. M. (2013). Balance between autophagic pathways preserves retinal homeostasis. Aging Cell 12 (3), 478–488. 10.1111/acel.12072 23521856 PMC3655122

[B101] Russo R. Varano G. P. Adornetto A. Nazio F. Tettamanti G. Girardello R. (2018). Rapamycin and fasting sustain autophagy response activated by ischemia/reperfusion injury and promote retinal ganglion cell survival. Cell Death Dis. 9 (10), 981. 10.1038/s41419-018-1044-5 30250019 PMC6155349

[B102] Schuck S. (2020). Microautophagy - distinct molecular mechanisms handle cargoes of many sizes. J. Cell Sci. 133 (17), jcs246322. 10.1242/jcs.246322 32907930

[B103] Sears N. C. Boese E. A. Miller M. A. Fingert J. H. (2019). Mendelian genes in primary open angle glaucoma. Exp. Eye Res. 186, 107702. 10.1016/j.exer.2019.107702 31238079 PMC10207284

[B104] Shadel G. S. Horvath T. L. (2015). Mitochondrial ROS signaling in organismal homeostasis. Cell 163 (3), 560–569. 10.1016/j.cell.2015.10.001 26496603 PMC4634671

[B105] Shen X. Ying H. Qiu Y. Park J. S. Shyam R. Chi Z. L. (2011). Processing of optineurin in neuronal cells. J. Biol. Chem. 286 (5), 3618–3629. 10.1074/jbc.M110.175810 21059646 PMC3030366

[B106] Shi R. Weng J. Zhao L. Li X. M. Gao T. M. Kong J. (2012). Excessive autophagy contributes to neuron death in cerebral ischemia. CNS Neurosci. Ther. 18 (3), 250–260. 10.1111/j.1755-5949.2012.00295.x 22449108 PMC6493486

[B107] Shim M. S. Liton P. B. (2022). The physiological and pathophysiological roles of the autophagy lysosomal system in the conventional aqueous humor outflow pathway: More than cellular clean up. Prog. Retin. Eye Res. 90, 101064. 10.1016/j.preteyeres.2022.101064 35370083 PMC9464695

[B108] Shim M. S. Nettesheim A. Dixon A. Liton P. B. (2021). Primary cilia and the reciprocal activation of AKT and SMAD2/3 regulate stretch-induced autophagy in trabecular meshwork cells. Proc. Natl. Acad. Sci. U. S. A. 118 (13), e2021942118. 10.1073/pnas.2021942118 33753495 PMC8020776

[B109] Shim M. S. Nettesheim A. Hirt J. Liton P. B. (2020). The autophagic protein LC3 translocates to the nucleus and localizes in the nucleolus associated to NUFIP1 in response to cyclic mechanical stress. Autophagy 16 (7), 1248–1261. 10.1080/15548627.2019.1662584 31476975 PMC7469449

[B110] Shin J. Y. Park H. J. Kim H. N. Oh S. H. Bae J. S. Ha H. J. (2014). Mesenchymal stem cells enhance autophagy and increase β-amyloid clearance in Alzheimer disease models. Autophagy 10 (1), 32–44. 10.4161/auto.26508 24149893 PMC4389879

[B111] Sirohi K. Chalasani M. L. Sudhakar C. Kumari A. Radha V. Swarup G. (2013). M98K-OPTN induces transferrin receptor degradation and RAB12-mediated autophagic death in retinal ganglion cells. Autophagy 9 (4), 510–527. 10.4161/auto.23458 23357852 PMC3627667

[B112] Sirohi K. Swarup G. (2016). Defects in autophagy caused by glaucoma-associated mutations in optineurin. Exp. Eye Res. 144, 54–63. 10.1016/j.exer.2015.08.020 26302410

[B113] Skov A. G. Rives A. S. Freiberg J. Virgili G. Azuara-Blanco A. Kolko M. (2021). Comparative efficacy and safety of preserved versus preservative-free beta-blockers in patients with glaucoma or ocular hypertension: A systematic review. Acta Ophthalmol. 100, 253–261. 10.1111/aos.14926 34128326

[B114] Stamer W. D. Acott T. S. (2012). Current understanding of conventional outflow dysfunction in glaucoma. Curr. Opin. Ophthalmol. 23 (2), 135–143. 10.1097/ICU.0b013e32834ff23e 22262082 PMC3770936

[B115] Stothert A. R. Fontaine S. N. Sabbagh J. J. Dickey C. A. (2016). Targeting the ER-autophagy system in the trabecular meshwork to treat glaucoma. Exp. Eye Res. 144, 38–45. 10.1016/j.exer.2015.08.017 26302411 PMC4698052

[B116] Su Q. Zheng B. Wang C. Y. Yang Y. Z. Luo W. W. Ma S. M. (2018). Oxidative stress induces neuronal apoptosis through suppressing transcription factor EB phosphorylation at Ser467. Cell. Physiol. biochem. 46 (4), 1536–1554. 10.1159/000489198 29689560

[B117] Swarup G. Sayyad Z. (2018). Altered functions and interactions of glaucoma-associated mutants of optineurin. Front. Immunol. 9, 1287. 10.3389/fimmu.2018.01287 29951055 PMC6008547

[B118] Tang L. H. C. Fung F. K. C. Lai A. K. W. Wong I. Y. H. Shih K. C. Lo A. C. Y. (2021). Autophagic upregulation is cytoprotective in ischemia/reperfusion-injured retina and retinal progenitor cells. Int. J. Mol. Sci. 22 (16), 8446. 10.3390/ijms22168446 34445152 PMC8395130

[B119] Tang R. H. Qi R. Q. Liu H. Y. (2019). Interleukin-4 affects microglial autophagic flux. Neural Regen. Res. 14 (9), 1594–1602. 10.4103/1673-5374.255975 31089059 PMC6557092

[B120] Teodorof-Diedrich C. Spector S. A. (2018). Human immunodeficiency virus type 1 gp120 and tat induce mitochondrial fragmentation and incomplete mitophagy in human neurons. J. Virol. 92 (22), 1–16. 10.1128/jvi.00993-18 PMC6206485 30158296

[B121] Tham Y. C. Li X. Wong T. Y. Quigley H. A. Aung T. Cheng C. Y. (2014). Global prevalence of glaucoma and projections of glaucoma burden through 2040: A systematic review and meta-analysis. Ophthalmology 121 (11), 2081–2090. 10.1016/j.ophtha.2014.05.013 24974815

[B122] Töteberg-Harms M. Meier-Gibbons F. (2021). Is laser trabeculoplasty the new star in glaucoma treatment? Curr. Opin. Ophthalmol. 32 (2), 141–147. 10.1097/icu.0000000000000732 33470670

[B123] Tran V. T. Ho P. T. Cabrera L. Torres J. E. Bhattacharya S. K. (2014). Mechanotransduction channels of the trabecular meshwork. Curr. Eye Res. 39 (3), 291–303. 10.3109/02713683.2013.842593 24215462

[B124] Tsai H. Y. Lai H. Chen Z. Y. Lin T. C. Khor W. Kuo W. C. (2022). Inhibition of DUSP6 activates autophagy and rescues the retinal pigment epithelium in sodium iodate-induced retinal degeneration models *in vivo* and *in vitro* . Biomedicines 10 (1), 159. 10.3390/biomedicines10010159 35052838 PMC8773272

[B125] Tucker B. A. Solivan-Timpe F. Roos B. R. Anfinson K. R. Robin A. L. Wiley L. A. (2014). Duplication of TBK1 stimulates autophagy in iPSC-derived retinal cells from a patient with normal tension glaucoma. J. Stem Cell Res. Ther. 3 (5), 161. 10.4172/2157-7633.1000161 24883232 PMC4038935

[B126] Vecino E. Rodriguez F. D. Ruzafa N. Pereiro X. Sharma S. C. (2016). Glia-neuron interactions in the mammalian retina. Prog. Retin. Eye Res. 51, 1–40. 10.1016/j.preteyeres.2015.06.003 26113209

[B127] Villarejo-Zori B. Jiménez-Loygorri J. I. Zapata-Muñoz J. Bell K. Boya P. (2021). New insights into the role of autophagy in retinal and eye diseases. Mol. Asp. Med. 82, 101038. 10.1016/j.mam.2021.101038 34620506

[B128] Wang R. (2017). Effect of acupuncture for intraocular pressure control and eye function in patients w ith glaucoma. J Int. Eye Sci. 17 (05), 958–960.

[B129] Wang Y. Huang C. Zhang H. Wu R. (2015). Autophagy in glaucoma: Crosstalk with apoptosis and its implications. Brain Res. Bull. 117, 1–9. 10.1016/j.brainresbull.2015.06.001 26073842

[B130] Wang Y. Lv J. Huang C. Li X. Chen Y. Wu W. (2021). Human umbilical cord-mesenchymal stem cells survive and migrate within the vitreous cavity and ameliorate retinal damage in a novel rat model of chronic glaucoma. Stem Cells Int. 2021, 8852517. 10.1155/2021/8852517 34733333 PMC8560304

[B131] Weinreb R. N. Khaw P. T. (2004). Primary open-angle glaucoma. Lancet 363 (9422), 1711–1720. 10.1016/s0140-6736(04)16257-0 15158634

[B132] Wen-bo H. Jun F. Jie C. Wei W. (2020). Effect of tiluo yangxue mingmu prescription on visual function, vessel density and thickness of retinal nerve fiber layer of optic nerve in glaucoma patients. J Chin. J. Exp. Traditional Med. Formulae 26 (24), 108–115. 10.13422/j.cnki.syfjx.20202213

[B133] Wise J. B. Witter S. L. (1979). Argon laser therapy for open-angle glaucoma: A pilot study. Arch. Ophthalmol. 97 (2), 319–322. 10.1001/archopht.1979.01020010165017 575877

[B134] Wolters J. E. J. van Mechelen R. J. S. Al Majidi R. Pinchuk L. Webers C. A. B. Beckers H. J. M. (2021). History, presence, and future of mitomycin C in glaucoma filtration surgery. Curr. Opin. Ophthalmol. 32 (2), 148–159. 10.1097/icu.0000000000000729 33315724

[B135] Wong E. Cuervo A. M. (2010). Autophagy gone awry in neurodegenerative diseases. Nat. Neurosci. 13 (7), 805–811. 10.1038/nn.2575 20581817 PMC4038747

[B136] Wong P. M. Puente C. Ganley I. G. Jiang X. (2013). The ULK1 complex: Sensing nutrient signals for autophagy activation. Autophagy 9 (2), 124–137. 10.4161/auto.23323 23295650 PMC3552878

[B137] Wong S. Q. Kumar A. V. Mills J. Lapierre L. R. (2020). Autophagy in aging and longevity. Hum. Genet. 139 (3), 277–290. 10.1007/s00439-019-02031-7 31144030 PMC6884674

[B138] Xirui Y. Jixue W. Feixue D. He S. (2021). The treatment of glaucoma with liver depression A systematic review and meta-analysis of randomized controlled trials. J J. Traditional Chin. Ophthalmol. 31 (03), 218–223. 10.13444/j.cnki.zgzyykzz.2021.03.016

[B139] Xu L. Wang X. Wu M. (2017). Topical medication instillation techniques for glaucoma. Cochrane Database Syst. Rev. 2 (2), Cd010520. 10.1002/14651858.CD010520.pub2 28218404 PMC5419432

[B140] Yan X. Wu S. Liu Q. Cheng Y. Zhang J. Wang N. (2022). Myocilin gene mutation induced autophagy activation causes dysfunction of trabecular meshwork cells. Front. Cell Dev. Biol. 10, 900777. 10.3389/fcell.2022.900777 35615698 PMC9124892

[B141] Yauger Y. J. Bermudez S. Moritz K. E. Glaser E. Stoica B. Byrnes K. R. (2019). Iron accentuated reactive oxygen species release by NADPH oxidase in activated microglia contributes to oxidative stress *in vitro* . J. Neuroinflammation 16 (1), 41. 10.1186/s12974-019-1430-7 30777083 PMC6378754

[B142] Yim W. W. Mizushima N. (2020). Lysosome biology in autophagy. Cell Discov. 6, 6. 10.1038/s41421-020-0141-7 32047650 PMC7010707

[B143] Zhang M. L. Zhao G. L. Hou Y. Zhong S. M. Xu L. J. Li F. (2020). Rac1 conditional deletion attenuates retinal ganglion cell apoptosis by accelerating autophagic flux in a mouse model of chronic ocular hypertension. Cell Death Dis. 11 (9), 734. 10.1038/s41419-020-02951-7 32913260 PMC7484783

[B144] Zhang Q. He C. Li R. Ke Y. Sun K. Wang J. (2021a). miR-708 and miR-335-3p inhibit the apoptosis of retinal ganglion cells through suppressing autophagy. J. Mol. Neurosci. 71 (2), 284–292. 10.1007/s12031-020-01648-y 32683666

[B145] Zhang S. Shao Z. Liu X. Hou M. Cheng F. Lei D. (2021b). The E50K optineurin mutation impacts autophagy-mediated degradation of TDP-43 and leads to RGC apoptosis *in vivo* and *in vitro* . Cell Death Discov. 7 (1), 49. 10.1038/s41420-021-00432-0 33723228 PMC7960725

[B146] Zhang W. Tian T. Gong S. X. Huang W. Q. Zhou Q. Y. Wang A. P. (2021c). Microglia-associated neuroinflammation is a potential therapeutic target for ischemic stroke. Neural Regen. Res. 16 (1), 6–11. 10.4103/1673-5374.286954 32788440 PMC7818879

[B147] Zhang Z. Yang X. Song Y. Q. Tu J. (2021d). Autophagy in Alzheimer's disease pathogenesis: Therapeutic potential and future perspectives. Ageing Res. Rev. 72, 101464. 10.1016/j.arr.2021.101464 34551326

[B148] Zubova S. G. Suvorova II Karpenko M. N. (2022). Macrophage and microglia polarization: Focus on autophagy-dependent reprogramming. Front. Biosci. 14 (1), 3. 10.31083/j.fbs1401003 35320914

